# Real-Time Monitoring of Tumorigenesis, Dissemination, & Drug Response in a Preclinical Model of Lymphangioleiomyomatosis/Tuberous Sclerosis Complex

**DOI:** 10.1371/journal.pone.0038589

**Published:** 2012-06-15

**Authors:** Fangbing Liu, Elaine P. Lunsford, Jingli Tong, Yoshitomo Ashitate, Summer L. Gibbs, Jane Yu, Hak Soo Choi, Elizabeth P. Henske, John V. Frangioni

**Affiliations:** 1 Division of Hematology/Oncology, Beth Israel Deaconess Medical Center, Harvard Medical School, Boston, Massachusetts, United States of America; 2 Longwood Small Animal Imaging Facility, Beth Israel Deaconess Medical Center, Harvard Medical School, Boston, Massachusetts, United States of America; 3 Division of Pulmonary, Critical Care and Sleep Medicine, Beth Israel Deaconess Medical Center, Harvard Medical School, Boston, Massachusetts, United States of America; 4 Division of Pulmonary and Critical Care Medicine, Brigham and Women's Hospital and Harvard Medical School, Boston, Massachusetts, United States of America; 5 Department of Radiology, Beth Israel Deaconess Medical Center, Harvard Medical School, Boston, Massachusetts, United States of America; Duke University Medical Center, United States of America

## Abstract

**Background:**

TSC2-deficient cells can proliferate in the lungs, kidneys, and other organs causing devastating progressive multisystem disorders such as lymphangioleiomyomatosis (LAM) and tuberous sclerosis complex (TSC). Preclinical models utilizing LAM patient-derived cells have been difficult to establish. We developed a novel animal model system to study the molecular mechanisms of TSC/LAM pathogenesis and tumorigenesis and provide a platform for drug testing.

**Methods and Findings:**

TSC2-deficient human cells, derived from the angiomyolipoma of a LAM patient, were engineered to co-express both sodium-iodide symporter (NIS) and green fluorescent protein (GFP). Cells were inoculated intraparenchymally, intravenously, or intratracheally into athymic NCr *nu/nu* mice and cells were tracked and quantified using single photon emission computed tomography (SPECT) and computed tomography (CT). Surprisingly, TSC2-deficient cells administered intratracheally resulted in rapid dissemination to lymph node basins throughout the body, and histopathological changes in the lung consistent with LAM. Estrogen was found to be permissive for tumor growth and dissemination. Rapamycin inhibited tumor growth, but tumors regrew after the drug treatment was withdrawn.

**Conclusions:**

We generated homogeneous NIS/GFP co-expressing TSC2-deficient, patient-derived cells that can proliferate and migrate *in vivo* after intratracheal instillation. Although the animal model we describe has some limitations, we demonstrate that systemic tumors formed from TSC2-deficient cells can be monitored and quantified noninvasively over time using SPECT/CT, thus providing a much needed model system for *in vivo* drug testing and mechanistic studies of TSC2-deficient cells and their related clinical syndromes.

## Introduction

Tuberous sclerosis complex (TSC) is a tumor suppressor gene syndrome and autosomal-dominant genetic disease with a prevalence of 1 in 6,000 births and a 95% penetrance [1], [2]. The characteristic manifestations of TSC include cortical tubers, subependymal giant cell astrocytomas, cardiac rhabdomyomas, renal angiomyolipoma (AML), and life-threatening pulmonary lymphangioleiomyomatosis (LAM) [3].

LAM is a rare disease affecting primarily women of childbearing age. Abnormal proliferation of smooth muscle-like cells (called LAM cells) leads to distortion of lung architecture, cystic lung destruction, and enlargement of the thoracic and abdominal axial lymphatics, rarely resulting in lymphedema [4], [5]. LAM occurs sporadically and is also found in 30% to 40% of adult female TSC patients [6]. Approximately two-thirds of women with sporadic LAM also have renal AML.

TSC and LAM are caused by mutations of one of two tumor suppressor genes, *TSC1* and *TSC2*, located on chromosome 9q34 or chromosome 16p13, respectively [7], [8], [9]. The heterodimer of hamartin (encoded by *TSC1*) and tuberin (*TSC2*) suppress the mammalian target of rapamycin (mTOR), a major regulator of cell cycle progression, cell growth, and proliferation [10], [11]. mTOR is a serine-threonine kinase that receives input from various signaling pathways to activate translation, thus increasing cell proliferation and growth [12]. TSC2 loss or mutation leads to disruption of the tuberin-hamartin heterodimer. Additionally, dysregulation of coordinated mTOR and PI3K signaling contributes to tumorigenesis, a common characteristic of LAM [13], [14], [15].

LAM and AML are comprised of cells with *TSC1* or *TSC2* mutations, which permits investigators to discriminate between “two hit” cells [16], [17] and adjacent cells or matrix. Lung LAM cells develop as nodular structures with spindle-shaped and epithelioid cells, which are immunophenotypically distinct. Although they both express alpha-smooth muscle actin (α-SMA), epithelioid cells often harbor melanoma markers such as gp100, MART-1 [18]. TSC2-deficient cells are histologically benign, but have the potential to metastasize *in vivo* [19], [20], [21]. Several TSC2-deficient or null cells have been isolated from AML patients previously, but isolating and establishing sustained cultures of AML and LAM cells has been challenging [22], [23], [24], in part because of the heterogeneity of LAM nodules [25]. Nevertheless, the need to obtain an understanding of how existing cell lines behave *in vivo* is of paramount importance.

There are several imaging modalities that can be used to monitor tumor metastasis and proliferation *in vivo*, including optical imaging, positron emission tomography (PET), single photon emission computed tomography (SPECT), X-ray computed tomography (CT), and magnetic resonance imaging (MRI). A variety of gene reporter systems have been used to enhance the sensitivity and specificity for tracking cells *in vivo* with these modalities. The sodium-iodide symporter (NIS), a mediator of iodide anion uptake, is mainly expressed on the basolateral membrane of thyroid follicular cells in the thyroid gland and superficial mucin-secreting epithelial cells in the stomach [26], [27]. Thus, ectopic expression of NIS on any other cell type permits sensitive detection by using a radiotracer that is recognized by the symporter. Fortunately, in addition to the iodide anion, NIS also mediates transport of the pertechnetate anion ^99m^TcO_4_^-^, providing safe, nonvolatile, inexpensive, and noninvasive tracking using SPECT [28], [29].

In this study, we engineered homogeneous TSC2-deficient LAM patient-derived cells that co-express NIS and GFP and studied their pattern of proliferation and dissemination *in vivo*, and their response to hormones and drugs.

## Methods

### Plasmids and Cell Lines

621-101 cells were previously isolated from a renal angiomyolipoma of a sporadic LAM patient and immortalized, as described previously [30], [31]. Generation and use of the 621-101 cells was approved by the Institutional Review Board at Fox Chase Cancer Center in Philadelphia, and written informed consent was obtained from the participant in the study. 621-101 cells were cultured in a 50/50 mixture of DMEM/F12 (Invitrogen, Carlsbad, CA) supplemented with epidermal growth factor (EGF, 10 ng/ml; Sigma-Aldrich, St. Louis, MO), 200-nM hydrocortisone (Sigma-Aldrich), 25-µg/ml insulin (Sigma-Aldrich), 50-nM sodium selenite (Sigma-Aldrich), 10-µg/ml transferrin (Sigma-Aldrich), 1.6-µM ferrous sulfate (Sigma-Aldrich), and 15% fetal bovine serum (Gemini) as described by Arbiser et al. [22].

Human solute carrier family member 5 (*SLC5A5*; sodium-iodide symporter) was purchased from Open Biosystems (Huntsville, AL). The verified cDNA of human NIS was cloned into the pBMN-GFP retroviral plasmid (Orbigen Inc., San Diego, CA). The detailed method for high titer virus production is described in the Methods S1. The freshly collected supernatants containing Retro-NIS/GFP viruses were used to infect 621-101 cells under selection with 2-mg/mL puromycin. After subcloning, the chosen puromycin-resistant cell line was designated 621-327 and multiple vials were frozen at the time of establishment. A single vial was expanded no more than 15 passages after thawing prior to inoculation into mice.

HEK293T cells (American Type Culture Collection, Manassas, VA) were grown with DMEM medium containing 10% fetal bovine serum, penicillin (100 U/mL), streptomycin (100 µg/mL), and 2-mM L-glutamine (Mediatech Inc., Manassas, VA).

### Immunofluorescence Microscopy and Immunohistochemistry

Immunofluorescence analysis of NIS expression in cells and tissues was performed using rabbit anti-NIS (1∶50; Abcam, Cambridge, MA) and rabbit monoclonal anti-TSC2, specifically recognizing C-terminal region of human TSC2/tuberin (1∶50; Millipore, Temecula, CA), respectively. Cy3 (Jackson ImmunoResearch, West Grove, PA) goat anti-rabbit IgG (H+L) (1∶200) was used as a secondary antibody. For immunohistochemistry, thin sections of frozen samples were blocked with 5% goat serum in PBS-T and incubated with rabbit anti-Ki-67 (1∶50, Santa Cruz), rabbit anti-NIS (1∶50, Abcam) or rabbit anti-GFP polyclonal antibody (1∶200; Abcam), respectively. Alexa Fluor 680 (Invitrogen, Carlsbad, CA) goat anti-rabbit IgG (H+L) (1∶200) was used as a secondary antibody. TUNEL staining was performed using a TACS^®^ 2 TdT-DAB *In Situ* Apoptosis Detection kit according to the manufacturer’s instructions (Trevigen, Inc., Gaithersburg, MD). Detailed methods are described in Methods S1.

### Immunoblotting

Cells were lysed with 0.5 ml of M-PER™ (Pierce, Rockford, IL) mammalian protein extraction reagent. Protein concentration was quantified using a Bradford kit and normalized to 20 µg per lane. Samples were boiled for 5 min and separated on a 4% to 20% Tris-Tricine SDS-PAGE gel. After transfer to PVDF membranes (PerkinElmer Life Science, Boston, MA) and blocking at room temperature for 2 h with 5% dried milk, membranes were incubated overnight at 4°C with antibodies against tuberin (C-20) (1∶1000; Santa Cruz), phospho-Akt (1∶1000; Cell Signaling), Akt (1∶1000; Santa Cruz), phospho-S6 (1∶1000; Cell Signaling), NIS (1∶1000; Abcam), GFP (1∶1000; Abcam) or β-actin (Cell Signaling). Membranes were washed, incubated for 1 h with the appropriate secondary antibodies (1∶10000; Thermo Scientific, Rockford, IL), and quantified using SuperSignal West Pico Chemiluminescent Substrate (Thermo Scientific).

### *In vitro* Radiotracer ^99m^TcO_4_ Uptake

621-327 and 621-101 cells were cultured in 6-cm dishes until 80% confluent and in log-phase of growth. Cells were washed once with 5 ml of prewarmed PBS before adding 250-µCi 99^m^Tc-pertechnetate in 3-ml PBS. Cells were incubated at 37°C for 1 h. After incubation, the cells were washed once with PBS before performing SPECT/CT to quantitate ^99m^Tc-pertechnetate uptake.

### TSC2-deficient Cell Dissemination and Proliferation in Animal Models

Animal studies were performed in accordance with the approved institutional protocol #155–2008 by the Institutional Animal Care and Use Committee (IACUC) of Beth Israel Deaconess Medical Center. Female and male athymic NCr *nu/nu* mice were purchased from Taconic Farms (Hudson, NY). At the time of tumor cell inoculation, mice averaged 5 to 6 weeks of age and weighed 22 g ±3 g. For tumor cell inoculation, anesthesia was induced using intraperitoneal (IP) injection of a mixture of 50-mg/kg ketamine hydrochloride (Ketaject; Phoenix Pharmaceutical Inc., St. Joseph, MO) and 5-mg/kg xylazine hydrochloride (Bayer Corp., Shawnee Mission, KS). Approximately 10 million TSC2-deficient cells in 100 µL PBS were administrated into female mice via (1) intravenous injection, (2) direct pulmonary injection, or (3) delivery of tumor cells intratracheally using a 1cc syringe and 20-gauge 1.5-inch needle. Detailed methods are described in Methods S1.

### SPECT/CT Imaging of TSC2-deficient Tumors in Mice

TSC2-deficient tumors were assessed on the days indicated using micro SPECT/CT. Two hours prior to imaging, animals were anesthetized with 2% isoflurane/balance O_2_ and 500 µCi of ^99m^TcO_4_^-^ in 50-µl saline, which was injected intravenously. For imaging, animals were anesthetized with 2% isoflurane/balance O_2_ and scanned on a NanoSPECT/CT (Bioscan Inc., Washington, DC). Quantitation of tumor volume, and ^99m^Tc uptake was performed using InVivoScope software (Bioscan Inc., Washington, DC). All imaging parameters are provided in Methods S1. Six to eight tumors in 1 mouse were quantified, and the average served as the output for statistical analysis.

**Figure 1:**
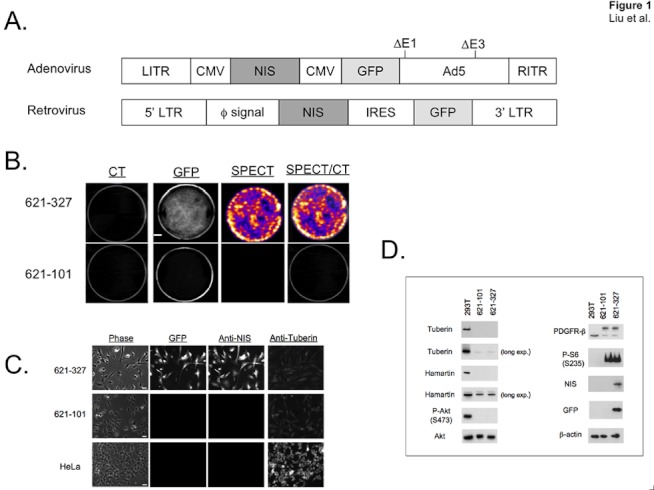
Generation and characterization of *in vivo* trackable TSC2-deficient cells. A. Expression cassettes of adenoviral (Ad-NIS/GFP) and retroviral (Retro-NIS/GFP) vectors. CMV  =  cytomegalovirus promoter, NIS  =  sodium-iodide symporter, LITR  =  left-handed inverted terminal repeat, RITR  =  right-handed inverted terminal repeat, GFP  =  green fluorescent protein, ΔE1, ΔE3 =  E1 and E3 deletions of adenovirus type 5 (Ad5) backbone sequence, 5′ LTR = 5′ long terminal repeat, φ signal  =  virus packing signal, IRES  =  internal ribosome entry site, 3′ LTR  = 3′ long terminal repeat. B. *In vitro* uptake of ^99m^Tc-pertechnetate and GFP fluorescence in stable Retro-NIS−/GFP-expressing 621-327 cells (top row) and control 621-101 cells (bottom row). Scale bar = 6 mm. C. Immunofluorescent detection of GFP, NIS, and tuberin in TSC2-deficient 621-327 cells (top row), control 621-101 cells (middle row), and HeLa cells (bottom row). GFP fluorescence (2nd column), staining with primary anti-NIS specific antibody (3rd column) or anti-tuberin antibody (right column) with secondary Cy3 antibody conjugates is shown along with phase contrast (left column). Scale bar = 50 µm. D. Western blot analysis of TSC2-expressing HEK293T control cells, and TSC2-deficient 621-101 cells and 621-327 cells, using antibodies to key signaling proteins, NIS, and GFP. A beta actin loading control is also shown, as are long exposure times (long exp.) for tuberin and hamartin.

### Identification of *TSC2* Mutations in Tumor Cells

TSC2-deficient tumors and organs were excised immediately after animal sacrifice, embedded in Tissue-Tek O.C.T, flash-frozen in LN_2_, and stored at −80°C until use. Genomic DNA was isolated using a QIAamp DNA Mini Kit (Qiagen GmbH, Hilden, Germany). For a positive control, genomic DNA was isolated from 621-101 or 621-327 cells. Genomic DNA from tumor-bearing mice was used as a template to amplify exon 17 of the *TSC2* gene. The PCR-amplified exon 17 products were verified by DNA sequencing. The detailed methods and primer sequences are described in Methods S1.

### Hormone and Drug Treatment Studies

For the hormone and sex study, male and female mice (n = 3 per group) were implanted with 17ß-Estradiol pellets (0.18 mg/pellet, 90 day release; Innovative Research, Sarasota, FL) or control placebo pellets subcutaneously. The release rate was 1.5 to 2 µg daily. Mice were inoculated with tumor cells by intratracheal instillation 7 to 10 days after pellet implantation. Radiotracer uptake of tumors was measured using SPECT/CT every 2 weeks after tumor cell inoculation. Prior to rapamycin (Santa Cruz Biotechnology, Santa Cruz, CA) treatment, 2 groups of n = 4 mice each were created so that baseline tumor size and number were equal 2 weeks post-tumor cell inoculation. One group was treated with 100 µL of intraperitoneal rapamycin (8 mg/kg) injection once daily for 4 weeks as described by Woodrum et al [32]. The control group received 100 µL of the vehicle (PBS). Tumor growth was quantified every other week as described above using SPECT.

**Figure 2:**
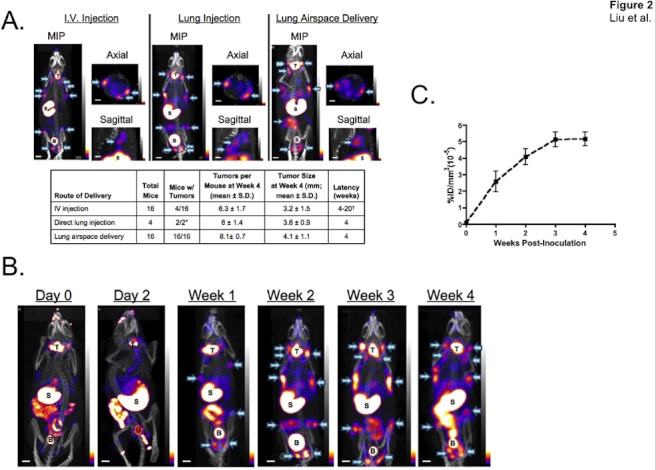
Development of a quantifiable LAM/TSC animal model system. A. SPECT/CT imaging of NCr *nu/nu* mice 4 weeks after inoculation of 621-327 cells by intravenous injection (left), intrapulmonary injection (middle), and intratracheal instillation (right). MIP  =  maximal intensity projection; T  =  thyroid; S  =  stomach; B  =  bladder. Arrows indicate tumors confirmed after animal sacrifice. Scale bars = 1 cm. *Two of four mice died immediately after direct lung injections. †One mouse showed radiotracer uptake at week 4; others varied from week 8 to week 20. B. SPECT/CT imaging (MIP) of a single mouse preinoculation (Day 0) and at varying times after intratracheal delivery of 621-327 cells. T  =  thyroid; S  =  stomach; B  =  bladder. Arrows indicate tumors. Scale bars = 1 cm. C. Kinetics of tumor growth measured using SPECT. Shown is %ID/mm^3^*10^−5^ (mean ± SD) in 6 tumors per mouse for n = 3 mice over time.

## Results

### Establishment of an *in vivo* Trackable TSC2-deficient Cell Line Co-expressing NIS and GFP

Adenoviral and retroviral gene delivery vectors (Figure 1A) were used to deliver NIS and GFP genes into human cell line 621-101, which was derived from a LAM-associated angiomyolipoma. After initial proof of principle using adenovirus expression, retrovirus expression was used exclusively. Retrovirus-transduced NIS/GFP cells were selected and subcloned. A stable cell line designated 621-327 was obtained. Clonal 621-327 cells were adapted to grow in DMEM/F12 medium supplemented with 15% fetal bovine serum, penicillin (100 U/mL), and streptomycin (100 µg/mL). No EGF or other additives were added into the cell culture medium. 621-327 cells characterized by simultaneous expression of GFP and NIS are capable of mediating radiotracer ^99m^TcO_4_^-^ uptake, while the control 621-101 cells were not (Figure 1B). After continuous subculturing of a vial of these frozen cells for over 6 months, the tested 621-327 cells showed stable high-level expression of functional NIS and GFP, but low-level expression of tuberin (Figure 1C), and retained homogeneous morphology.

**Figure 3:**
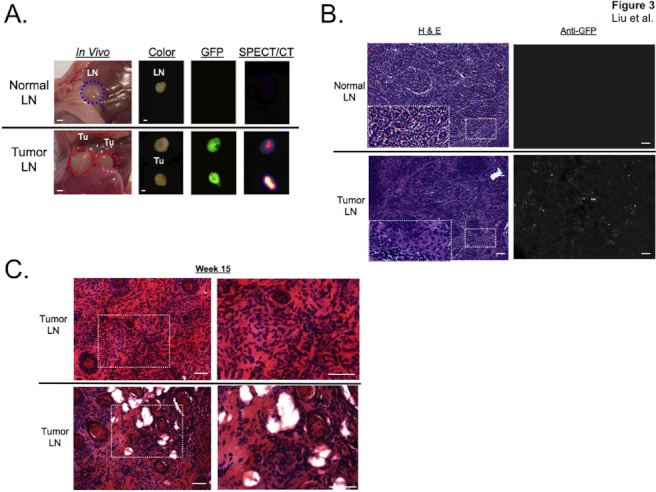
Lymph node metastasis and invasion by TSC2-deficient cells. A. Normal lymph node (LN; top row) and lymph node identified by GFP and NIS expression (bottom row) 4 weeks after intratracheal administration of 621-327 cells. Left column shows *in vivo* color video image. Right columns show same nodes *ex vivo* after resection, placement on black paper, and imaging using color video, GFP fluorescence, and SPECT/CT, respectively. Scale bars = 1 mm. B. Hematoxylin and eosin (H&E) staining of frozen sections from normal (top row) and tumor-infiltrated (bottom row) lymph nodes 2 weeks after intratracheal administration of 621-327 cells. Dotted rectangle inset  =  higher magnification. Consecutive tissue sections were also stained with anti-GFP antibody. Scale bars = 50 µm. C. H&E staining of paraffin-embedded, tumor-infiltrated lymph nodes at 15 weeks post-administration of 621-327 cells. Dotted rectangle inset  =  higher magnification. Scale bars = 50 µm.

### Characterization of TSC2-deficient Cells

To determine whether immortalized 621-101 and clonal 621-327 cells retained the functional consequences of TSC2 loss, protein levels of tuberin and hamartin and several relevant mTOR effector proteins were examined by Western blot analysis (Figure 1D). Tuberin and hamartin protein levels were dramatically reduced in both cell lines and the level of phospho-S6 (S235) was markedly elevated. Levels of PDGFR-b and phospho-Akt (S473) were decreased in TSC2-deficient cells compared to TSC-expressing HEK293T cells. These signaling changes are in agreement with previous findings in LAM cells, specifically that a *TSC2* loss or mutation leads to disruption of the tuberin-hamartin heterodimer resulting in activation of the mTOR signaling pathway, and dysregulation of S6K1 activation. Genomic DNA sequencing of 621-327 cells confirmed the nucleotide change G1832A in TSC2 cDNA on chromosome 16 (Figure S3), which results in an Arg611Gln missense mutation, and loss of heterozygosity of the TSC2 allele containing the wild-type residue (G).

**Figure 4:**
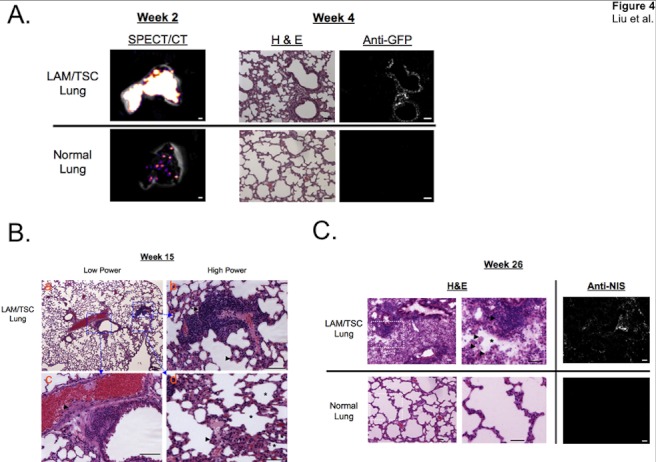
Proliferation and pathogenesis of TSC2-deficient cells: pulmonary LAM like-nodules and cysts. A. *Ex vivo* SPECT/CT imaging and antibody and H&E staining of lung. Resected whole lungs (left column) from LAM/TSC-bearing mice (top row) or control mice (bottom row) at 2 weeks postadministration of 621-327 cells. Scale bar = 1 mm. Also shown are H&E staining (middle column) and anti-GFP antibody staining (right column) of frozen sections from lungs at 4 weeks postadministration of 621-327 cells. Scale bars = 50 µm. B. H&E staining of paraffin-embedded lung tissue 15 weeks postadministration of 621-327 cells (top row) at low (top left) and high (top right and bottom row of dotted rectangles) magnification. Scale bars = 50 µm. C. Same as (B) except frozen sections at 26 weeks postadministration of 621-327 cells (left) with anti-human NIS antibody staining (right). Scale bars = 50 µm.

### TSC2-deficient Cell Dissemination and Proliferation in Orthotopic Mouse Models

LAM is an unusual disease because the tumor cells have a benign histological phenotype, but also have metastatic potential. To determine whether TSC2-deficient 621-327 cells are capable of disseminating and metastasizing *in vivo*, we administrated cells into mice by intravenous injection, direct lung injection, or intratracheal instillation. Interestingly, similar tumor cell deposition patterns were seen (Figure 2A), demonstrating that the TSC2-deficient cells *in vivo* retained tumorigenicity and were disseminated systemically, although the tumor take rate and latency varied in different administrations. Intravenous administration of cells into athymic NCr *nu/nu* mice resulted in a long latency and low take rate, with tumors also trending toward a smaller size compared with other modes of administration (Figure 2A). Direct needle injection of cells into the lung resulted in distant tumor growth, but also high mortality. Delivery of 621-327 cells via intratracheal instillation resulted in tumor growth in 16 of 16 animals, short latency, widespread dissemination, and relatively large tumors.

The kinetics of tumor spread from the lung to the body is shown in Figure 2B. By day 2, signal in the lung was equivocal, likely due to respiratory motion (discussed below). Surprisingly, within 1 week of intratracheal administration, distinct radiotracer accumulation could be seen in the neck, axilla, groin region, right and/or left flanks, and lung (sagittal) in SPECT/CT images (Figures 2A and 2B, Video S1). Radiotracer uptake was also seen in the thyroid, salivary glands, and stomach, as endogenous NIS is expressed in these tissues.

Tumor size measured by ^99m^TcO_4_^-^ uptake was linearly proportional to that measured *ex vivo* using calipers, although consistently 2-fold larger (Figure S1). From weeks 1 to 3 after intratracheal administration, TSC2-deficient tumors increased in size. From week 4 onward, tumor size was relatively constant, and occasionally tumors at week 4 were slightly smaller than in week 3 (Figures 2B and 2C). To date, after analysis of 347 tumors in 55 animals, none have fully regressed after peaking at week 3 (data not shown).

**Figure 5:**
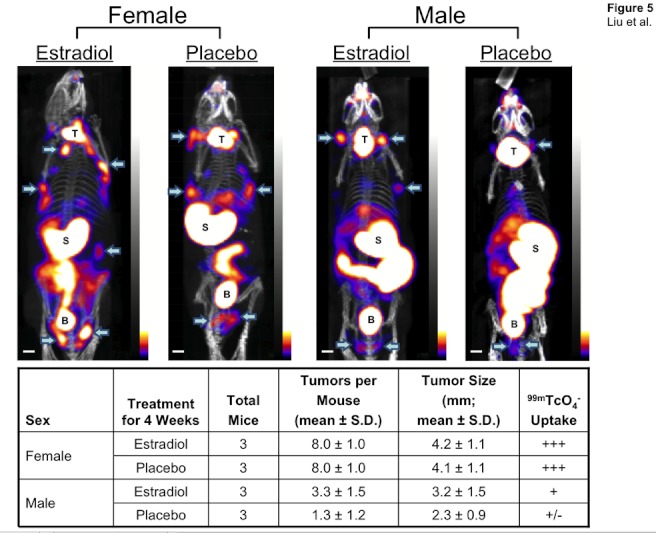
The effect of sex and exogenous estrogen on TSC2-deficient tumor growth. Female (left) or male (right) mice were implanted with 0.18 mg 17ß-estradiol pellets or control placebo pellets subcutaneously. Ten days later, 621-327 cells were administered intratracheally and tumors quantified every other week by SPECT/CT. Shown are the results after 4 weeks of hormone treatment. T  =  thyroid; S  =  stomach; B  =  bladder. Arrows indicate tumors. Scale bars  = 1 cm. +/−  = 0.3–1.4; +  = 1.5 - 2.5; ++  = 2.6–3.7; +++  =  ≥3.8%ID/mm^3^ (×10^−5^).

**Figure 6:**
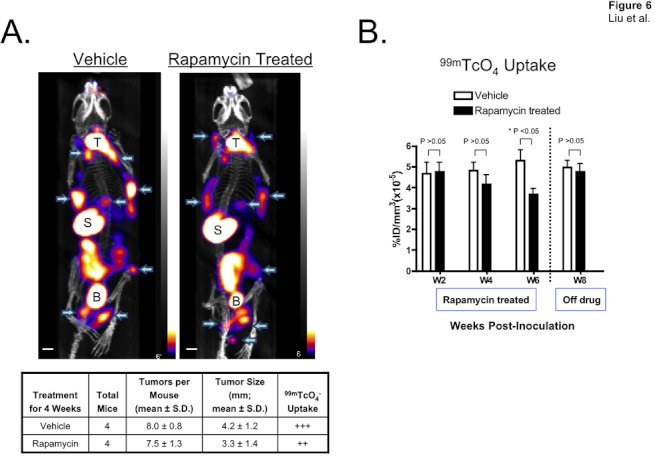
Quantitation of LAM/TSC tumor response to drug treatment. A. Typical SPECT/CT images of mice treated with rapamycin or vehicle for 4 weeks (i.e., 6 weeks after intratracheal administration of 621-327 cells). T  =  thyroid; S  =  stomach; B  =  bladder. Arrows indicate tumors. Scale bars = 1 cm. B.^ 99m^Tc-pertechnetate uptake in LAM/TSC tumors before, during, and after treatment with rapamycin or vehicle control. 621-327 cells were administered intratracheally at time = 0. Rapamycin treatment was during weeks 2 to 6. Mice were followed an additional 2 weeks off drug.

Resection of tissue with high radiotracer uptake (Figure S2) revealed a pattern consistent with lymphatic or hematogenous spread from the lung to lymph node basins. Indeed, histopathological analysis of lymph nodes at early time points after intratracheal administration revealed NIS−/GFP-expressing tumor deposits within lymph nodes (Figure 3A). Early tumor nodules were often seen within semi-encapsulated structures in lymph nodes (Figure 3B). By weeks 3 to 4, most lymph nodes analyzed were completely obliterated by the tumor cells with abundant or medium cytoplasm (arrow head). Interestingly, by 15 weeks postinoculation, some tumor cells developed a smooth muscle cell morphology, and some lymph nodes developed cystic or fluid-filled structures, although these cysts are not histologically identical to those observed in human LAM (Figure 3C). The weak SPECT signal measured in living mouse lungs (Figure 2A), and the strong signal seen after sacrifice (Figure 4A) suggest that the signal-to-noise ratio in the lungs was significantly degraded by respiratory motion.

### Formation of Pulmonary Abnormal Nodules in the Lungs

LAM is characterized by abnormal proliferation of smooth muscle like cells, leading to destruction of the lung architecture and formation of pulmonary cysts. To track the proliferation and differentiation of TSC2-deficient cells in the lung, we monitored and examined pathological changes of the mouse lungs at variable time points for half a year. Analysis of resected specimens revealed widespread tumor deposition and histopathological changes (Figures 4A and 4B). At early time points, the tumor cells appeared in small clusters adjacent to the lymphatic walls in the lungs, gathering against the thin wall of bronchioles, in capillary vessels, and in lymphatics (Figure 4A). Subsequently, pulmonary nodules of different sizes were formed in mouse lungs (Figures 4B and 4C). TSC2-deficient cells typically proliferated interstitially, between blood vessels and alveoli. Thin walled microscopic cystic areas were seen surrounding the tumor nodules (Figure 4C). Of note, there was considerable heterogeneity in the lungs of the mice, with some areas of the lung resembling the normal controls.

### Molecular and Genetic Characterization of TSC2-deficient Cells in Systemic Tumors

Immediately after SPECT/CT imaging at weeks 1, 2, 3, and 4 postinoculation, the major organs (lungs, heart, liver, spleen, kidneys) and lymph nodes were collected and dissected. To confirm that these cells were of human origin, we performed a nested polymerase chain reaction (PCR) using human TSC2 exon 17-specific primers and purified genomic DNA as templates. Because they were derived from a LAM-associate AML, the 621-327 cells have a specific TSC2 mutation (Figure S3), which does not occur in mouse cells. After DNA sequencing of PCR products, we identified this G1832A mutation of TSC2 exon17 in samples of the lungs, kidneys, and lymph nodes (Figure S4).

### Estrogen Enhances TSC2-deficient Cell Dissemination and Growth in Male Mice

LAM occurs almost exclusively in women, with onset usually during the childbearing years, which suggests that estrogen may play a role in disease progression. To measure the effect of estrogen, we implanted male and female mice with estradiol pellets or placebo pellets 7 to 10 days before inoculation of tumor TSC2-deficient cells. When assessed at 4 weeks, the 621-327 cells had a much lower initiation and growth rate in male mice, and lower overall radiotracer accumulation, which was partially corrected by estradiol supplementation (Figure 5). Significant radiotracer uptake was seen in female mice implanted with either estradiol or placebo, suggesting that endogenous levels of estrogen in female mice are sufficient for tumor initiation and proliferation.

### Quantifying Drug Efficacy in Mice Bearing TSC2-deficient Tumors

We next investigated whether this orthotopic model would prove useful in quantifying tumor response to drug treatment. We confirmed that rapamycin does not affect ^99m^TcO_4_^-^ uptake by NIS (Figure S5A and B). Two weeks postimplantation, tumor-bearing mice with a similar baseline of tumor burden and tumor size were treated daily for 4 weeks with intraperitoneal injection of either 8-mg/kg rapamycin or phosphate buffered saline (n = 4 mice per group). Tumor size and location were quantified by SPECT/CT every other week. As shown in Figure 6A and B, mice treated with rapamycin for at least 4 weeks exhibited a statistically significant decrease in tumor uptake of radiotracer, which rebounded to pretreatment levels 2 weeks after drug treatment was stopped. Mice sacrificed at the end of this experiment showed no significant difference in apoptosis (Figure S6A) or proliferation (Figure S6B) between rapamycin treated and untreated tumors, confirming the stability of the tumor mass after treatment ended.

## Discussion

TSC2-deficient cells, exhibiting loss of TSC2 and hyperactivation of the mTORC1 signaling pathway, can proliferate and “metastasize,” yet lack many features of malignant transformation. Angiomyolipomas and LAM cells from patients with sporadic LAM are histologically identical and consistent with the hypothesis that these diseases share common genetic and pathogenetic mechanisms [33], [34]. Unfortunately, TSC genetic mouse models [35], [36] and other TSC models [37] do not develop lesions that resemble human pulmonary LAM [38].

The isolation of homogeneous TSC2-deficient cells is critical for developing robust and reliable animal models, yet has been challenging. Recently, two TSC2-null cell lines (TSC2^−/−^, TSC2^−/meth^) were isolated and characterized [23], [24]. Despite different genetic mutations, both TSC2^−/−^ and TSC2^−/meth^ smooth muscle-like cells shared proliferative and biochemical characteristics, including a requirement for epidermal growth factor (EGF), which cannot be replaced by insulin like-growth factor 1(IGF1) [23], [39]. In contrast, some human TCS2-deficient cells (e.g., 621-327) do not require supplemental EGF in cell culture medium for proliferation. Interestingly, the human TSC2-deficient, non-EGF requiring cells we obtained displayed tumorigenicity, are capable of disseminating from the lung to lymph nodes *in vivo*, and developed abnormal nodules in the lungs. To the best of our knowledge, this is the first time that a homogeneous LAM cell line capable of recapitulating some of the features of human LAM/TSC in animals has been successfully obtained from an angiomyolipoma with *TSC2* gene mutations.

The orthotopic model we describe, harboring inactivation of both alleles of TSC2, recapitulated several features of LAM. 621-327 cells were able to traverse the lung epithelium and to travel to distant tissues. Tumors in the lungs often had a morphology resembling smooth muscle cells and cystic structures were often present (Figure 3C). The migration of LAM cells to the lungs and lymphatics is one of the disease’s most distinctive characteristics. We found that 621-327 cells translocated from the lung to distinct lymph node basins very rapidly (within days). Although recent evidence suggests that human LAM might result in chyle-filled lymphangioleiomyomas of the axial lymphatics [4], [5], it should be noted that our animal model appears to display less central lymphatic involvement than expected. Nevertheless, it should now be possible to quantify the contribution of VEGF-D and lymphatic endothelial cells to this process. While many tumor models have focused on blood capillary angiogenesis, this model may permit the elucidation of basic mechanisms underlying lymph node migration and tumor-initiated lymphangiogenesis.

Orthotopic tumor models tend to better mimic the vascular, lymphatic, and stromal microenvironment of human tumors; therefore, they offer theoretical advantages over subcutaneous xenografts. Because the model we describe requires margination of cells across the lung epithelium, and displays preferential homing to a variety of tissues and organs, it will likely prove useful in understanding the molecular mechanisms underlying these different processes. For example, we have shown a male/female difference in tumor establishment, and the ability of estrogen to slightly increase tumor number and size in male mice. These findings are consistent with those from an Eker rat model [40], [41], as well as collective studies showing that LAM cell metastasis may be modulated by VEGF-D, matrix metalloproteinases, extracellular matrix, and estradiol [42], [43], [44], [45]. A proposed multistep model of lymphatic and estrogen-associated metastasis of LAM progenitor cells to lymph nodes, kidneys, and lungs was recently proposed [46].

A key feature of our model, with potential application to many other human tumor syndromes, is the use of SPECT to quantify tumor location and cell number in living animals. It should be noted that initial optimization of the model would not have been possible without SPECT because the tumors were small and looked very similar to lymph nodes (Figure 3A), which were not visible by CT. The use of the radioactive pertechnetate anion over iodide is advantageous in terms of cost and logistics, with the former being nonvolatile, readily available, and inexpensive. Although we used CT for anatomical referencing, future use of magnetic resonance imaging (MRI) will reduce overall radiation exposure and improve soft tissue contrast. Respiratory gating of the SPECT signal should also improve detectability in the lung. Co-expression of GFP provides a convenient, nonradioactive surrogate for NIS expression, although this was not employed in this study for full body imaging due to the high absorption and scatter of visible light.

The new model we describe, however, has significant limitations. Secondary mutations in the originating cell line may have occurred during passage. The location of cellular metastases does not match exactly the distribution seen in human LAM and TSC. For example, lymph node involvement is more peripheral in our model, and more central in LAM. After an initial burst of exponential growth, tumors stabilized in size for long periods of time. Whether this is due to the incompletely suppressed immune system of athymic NCR *nu/nu* mice is currently unknown.

Despite these limitations, from a practical standpoint, this novel animal model may prove useful in testing new drugs, and more importantly, drug combinations for the treatment of LAM/TSC, allowing the kinetics of tumor development and metastasis to be monitored over time. Recently, the mTOR inhibitor everolimus was approved for the treatment of slow-growing TSC tumors in the CNS [47], and its predecessor sirolimus was shown to modulate AML and LAM [48], [49]. However, tumor volume reduction required months of continuous treatment and the tumors regrew upon discontinuation of the treatment. A SPECT/CT model of LAM/TSC will permit quantifiable *in vivo* testing of drug combinations, and if combined with an *in vitro* prescreen, could result in an efficient, cost-saving, high-throughput drug-testing platform. The model also provides a platform for developing drugs that interfere with various steps in the progression of LAM/TSC. For example, administration of drugs preinoculation and up to 1 week postinoculation could target cell margination and metastasis. Drugs given from weeks 1 to 3 could target tumor cell proliferation, and drugs give from week 4 onward could target established tumors.

## Supporting Information

Figure S1 **Correlation of tumor size measured by SPECT (ordinate) and calipers (abscissa).**
(TIFF)

Figure S2 **SPECT/CT imaging of tumor mice (in vivo) and resected lymph nodes (ex vivo).** Two weeks after tumor inoculation, tumor bearing mice (in vivo) and dissected lymph nodes (ex vivo) were scanned with SPECT/CT. Lymph nodes exhibiting high radiotracer uptake and normal control lymph nodes were resected.
(TIFF)

Figure S3 **Genetic characterization of 621-327 cells.** Detection of the known TSC2 mutation (arrows; nucleotide change G1832A in TSC2 cDNA or G26266A in exon 17 on chromosome 16), which results in an Arg611Gln missense mutation.
(TIFF)

Figure S4 **Molecular and genetic characterization of tumor cells in vivo.** The characteristic G1832A TSC2 exon 17 mutation (arrows) from human 621-327 cells was found within the mouse lymph nodes (LN), lung, and kidney. The mutation (arrows; nucleotide change G1832A in TSC2 cDNA or G26266A in exon 17 on chromosome 16) results in an Arg611Gln missense mutation, and the fact that no wild-type allele is detected reflects loss of heterozygosity of the TSC2 allele containing the wild-type residue (G).
(TIFF)

Figure S5 **Effect of rapamycin on radiotracer uptake of NIS** ***in vitro*** **.** Conditions were (1) preincubate with 10 nM rapamycin for 24 h then 250 µCi ^99m^TcO_4_^-^ for 1 h prior to measurement, (2) preincubate with 10 nM rapamycin for 1 h then 250 µCi ^99m^TcO_4_^-^ for 1 h prior to measurement, and (3) Control with no rapamycin then 250 µCi ^99m^TcO_4_^-^ for 1 h prior to measurement. A) Numerical values (%ID/mm^3^×10^−5^; mean ± S.D.) and B) representative planar radioscintigraphic imaging of cell wells.
(TIFF)

Figure S6 **Apoptosis and cell proliferation of lung tumors 2 w after rapamycin treatment.** A. *In situ* apoptosis detection of lung tissues counterstained with Methyl Green. The frozen sections from rapamycin-treated or vehicle-treated lungs (top row), and the normal lung treated with TACS^®^-Nuclease for positive control (bottom left) and normal lung (normal control, bottom right). Arrow indicates an apoptotic cell. Scale bars = 50 µm. B. Detection of the cell proliferation marker Ki-67 in the lungs. Hematoxylin and eosin (H&E) staining of frozen sections from rapamycin or vehicle-treated lungs (top row). The consecutive tissue sections were also stained with Ki-67 antibody (bottom row). Scale bars = 50 µm.
(TIFF)

Video S1 **In Vivo whole body tracking of LAM/TSC tumors by SPECT/CT.** 360° rotation of a 3-D SPECT/CT reconstruction of a mouse 4 weeks after intratracheal administration of 621-327 cells. T  =  thyroid; S  =  stomach; B  =  bladder. Arrows indicate tumors.
(MOV)

Method S1 (DOC)
