# Supplementary figures and images for: Real-Time Monitoring of Tumorigenesis, Dissemination, & Drug Response in a Preclinical Model of Lymphangioleiomyomatosis/Tuberous Sclerosis Complex
Source: PLoS One. 2012 Jun 15;7(6):e38589. doi: 10.1371/journal.pone.0038589 (PMC3376142; doi:10.1371/journal.pone.0038589)

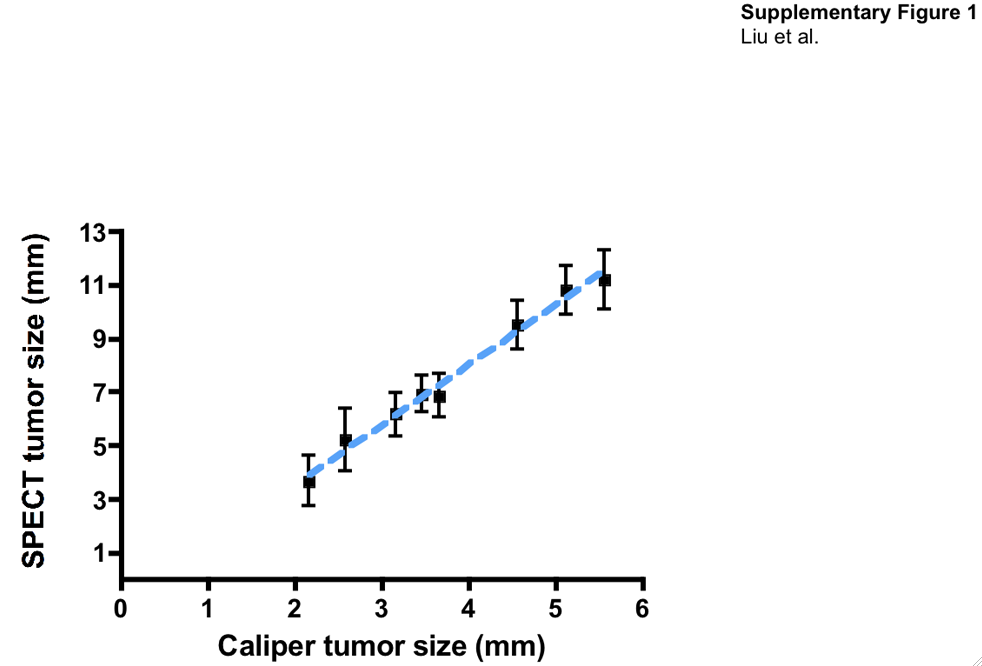

Supplement: Figure S1 — Correlation of tumor size measured by SPECT (ordinate) and calipers (abscissa). (TIFF) [file pone.0038589.s001.tiff]

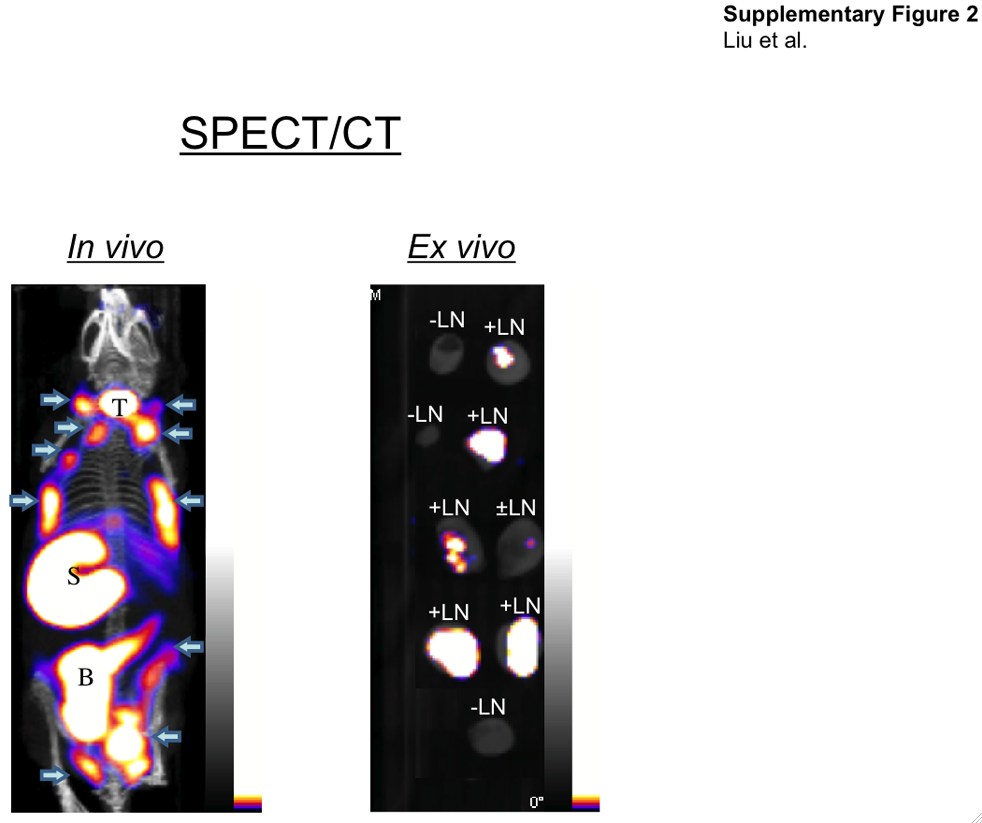

Supplement: Figure S2 — SPECT/CT imaging of tumor mice (in vivo) and resected lymph nodes (ex vivo). Two weeks after tumor inoculation, tumor bearing mice (in vivo) and dissected lymph nodes (ex vivo) were scanned with SPECT/CT. Lymph nodes exhibiting high radiotracer uptake and normal control lymph nodes were resected. (TIFF) [file pone.0038589.s002.tiff]

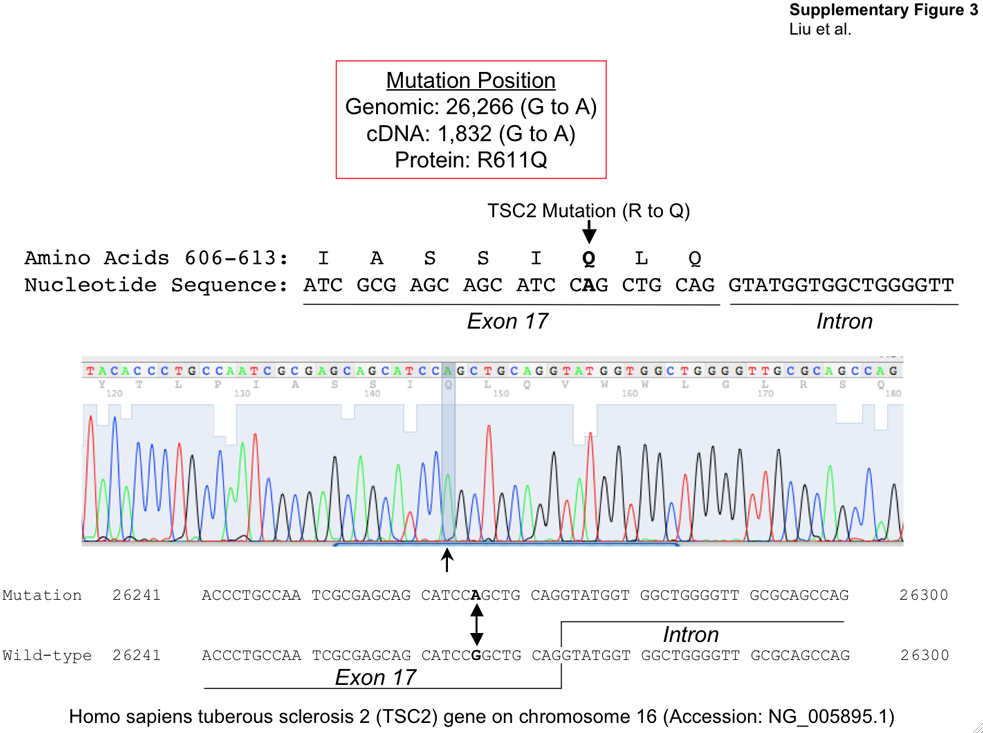

Supplement: Figure S3 — Genetic characterization of 621-327 cells. Detection of the known TSC2 mutation (arrows; nucleotide change G1832A in TSC2 cDNA or G26266A in exon 17 on chromosome 16), which results in an Arg611Gln missense mutation. (TIFF) [file pone.0038589.s003.tiff]

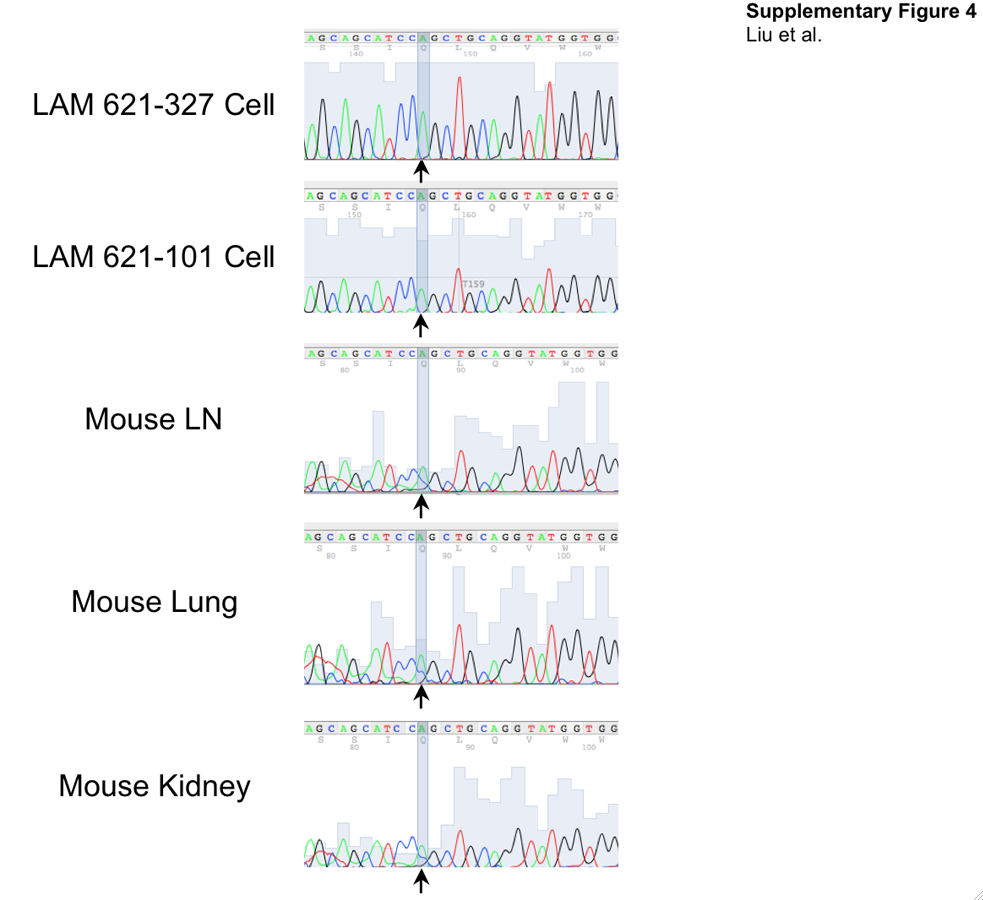

Supplement: Figure S4 — Molecular and genetic characterization of tumor cells in vivo. The characteristic G1832A TSC2 exon 17 mutation (arrows) from human 621-327 cells was found within the mouse lymph nodes (LN), lung, and kidney. The mutation (arrows; nucleotide change G1832A in TSC2 cDNA or G26266A in exon 17 on chromosome 16) results in an Arg611Gln missense mutation, and the fact that no wild-type allele is detected reflects loss of heterozygosity of the TSC2 allele containing the wild-type residue (G). (TIFF) [file pone.0038589.s004.tiff]

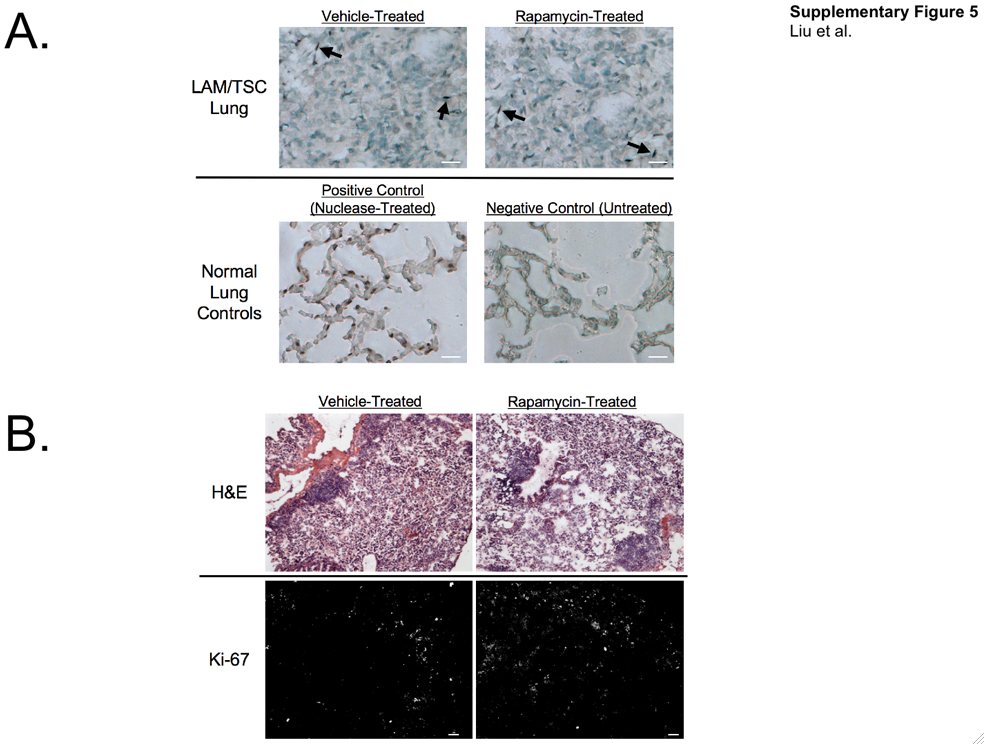

Supplement: Figure S5 — Effect of rapamycin on radiotracer uptake of NIS in vitro . Conditions were (1) preincubate with 10 nM rapamycin for 24 h then 250 µCi 99mTcO4 - for 1 h prior to measurement, (2) preincubate with 10 nM rapamycin for 1 h then 250 µCi 99mTcO4 - for 1 h prior to measurement, and (3) Control with no rapamycin then 250 µCi 99mTcO4 - for 1 h prior to measurement. A) Numerical values (%ID/mm3×10−5; mean ± S.D.) and B) representative planar radioscintigraphic imaging of cell wells. (TIFF) [file pone.0038589.s005.tiff]

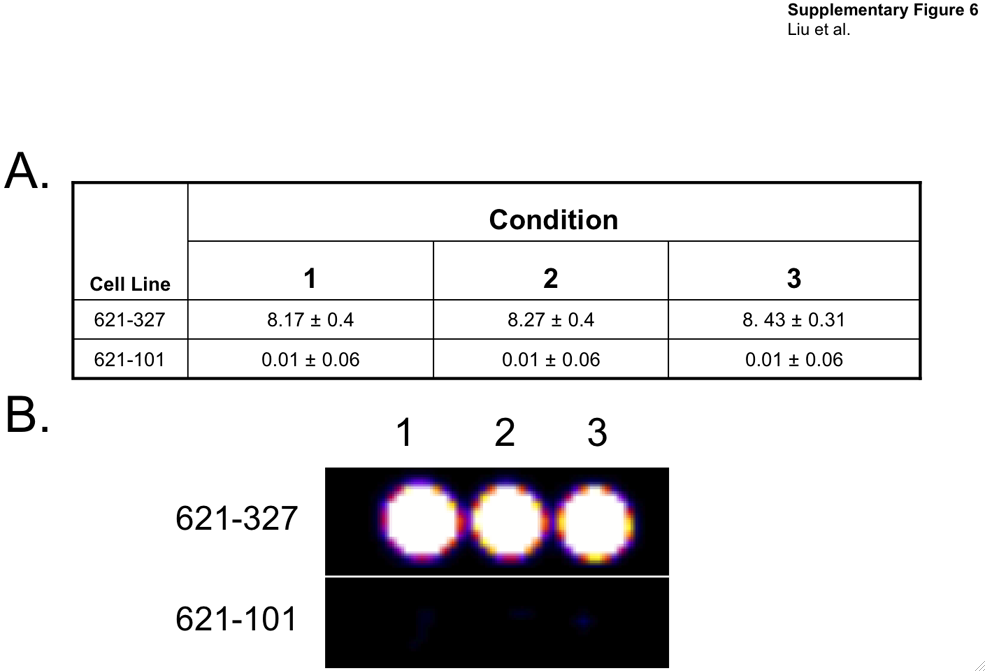

Supplement: Figure S6 — Apoptosis and cell proliferation of lung tumors 2 w after rapamycin treatment. A. In situ apoptosis detection of lung tissues counterstained with Methyl Green. The frozen sections from rapamycin-treated or vehicle-treated lungs (top row), and the normal lung treated with TACS®-Nuclease for positive control (bottom left) and normal lung (normal control, bottom right). Arrow indicates an apoptotic cell. Scale bars = 50 µm. B. Detection of the cell proliferation marker Ki-67 in the lungs. Hematoxylin and eosin (H&E) staining of frozen sections from rapamycin or vehicle-treated lungs (top row). The consecutive tissue sections were also stained with Ki-67 antibody (bottom row). Scale bars = 50 µm. (TIFF) [file pone.0038589.s006.tiff]
