# Supplementary material for: Real-Time Monitoring of Tumorigenesis, Dissemination, & Drug Response in a Preclinical Model of Lymphangioleiomyomatosis/Tuberous Sclerosis Complex
Source: PLoS One. 2012 Jun 15;7(6):e38589. doi: 10.1371/journal.pone.0038589 (PMC3376142; doi:10.1371/journal.pone.0038589)
Supplement: Method S1 — (DOC) [file pone.0038589.s008.doc]

**SUPPLEMENTARY METHODS**

**Real-Time Monitoring of Tumorigenesis, Dissemination, & Drug Response in a**

**Preclinical Model of Lymphangioleiomyomatosis**/**Tuberous Sclerosis Complex**

Fangbing Liu^1^, Elaine P. Lunsford^2^, Jingli Tong^3^, Yoshitomo Ashitate^1^,

Summer L. Gibbs^1^, Jane Yu^4^, Hak Soo Choi^1^, Elizabeth P. Henske^4^,

and John V. Frangioni^1,5,*^

^1^ Division of Hematology/Oncology, ^2^ Longwood Small Animal Imaging Facility, ^3^ Division of Pulmonary, Critical Care and Sleep Medicine, and ^5^ Department of Radiology, Beth Israel Deaconess Medical Center, Harvard Medical School, Boston, MA 02215

^4^ Division of Pulmonary and Critical Care Medicine, Brigham and Women's Hospital and Harvard Medical School, Boston, Massachusetts 02115, USA.

^*^To whom correspondence should be addressed. E-mail: jfrangio@bidmc.harvard.edu.

**The PDF file includes:**

Supplementary Methods

Supplementary References

**SUPPLEMENTARY METHODS**

**Plasmids:** Solute carrier family member 5 (*SLC5A5*; sodium-iodide symporter) was purchased from Open Biosystems (Huntsville, AL). The coding sequence of human sodium-iodide symporter (NIS) was cloned via EcoRI into mammalian expression vector pC. The orientation of the NIS insert in vector pC was analyzed by restriction enzyme digestions and verified by DNA sequencing.

**High titer adenoviral and retroviral GFP/NIS production and cell transduction:** cDNA of NIS from cloning vector pC was subcloned into pAdTrack-CMV via KpnI and NotI sites. pAdTrack-CMV also co-expresses GFP, which can be used as a convenient surrogate for adenovirus infection and gene transduction. The adenovirus packaging and production was followed by the pAdEasy system as described by Luo et al. [1]. Human angiomyolipoma-derived 621-101 cells [2] grown at 70% to 80% confluence on 10-cm diameter tissue culture plates or on cover slips in 12-well plates, were washed once with DMEM/F12, incubated with AdGFP/NIS adenovirus at a multiplicity of infection (MOI) of 1000:1 for 2 h, then returned to DMEM/F12 containing 20% fetal bovine serum (FBS; Gemini Bio-products, Calabasas, CA) for 48 h.

For retrovirus production, the cDNA of NIS was cloned into the BamHI-Notl sites of retroviral plasmid pBMN-GFP (Orbigen Inc., San Diego, CA) and packaged in 293 cells according to the product manuals. The supernatant containing Retro-GFP/NIS viruses were collected 48 to 72 h post-transfection. After centrifugation and filtration to remove any detached packaging cells, the freshly collected supernatants were used for infection of 621-101 cells in the presence of 8-µg/ml polybrene overnight. The selection of cells infected with retroviral particles containing the NIS gene and puromycin resistance gene (under the control of the CMV promoter and stable integration of the viral DNA into the host genome), were treated with 2-mg/ml puromycin for 3 weeks until resistant cell lines were established. Single clones were subcloned twice to obtain the final stable clones.

**Immunofluorescence microscopy and immunohistochemistry**: For analysis of cellular NIS and tuberin expression, 621-327 cells or control 621-101 cells were seeded at 3x10^4^ onto glass cover slips in 12-well plates and cultured for 24 h in DMEM/F12 containing 20% FBS, penicillin (100 U/mL), and streptomycin (100 µg/mL). The 621-101 cells were then infected with AdGFP/NIS at an MOI of 10^3^ and 0 (mock) for 24 h. Cells were fixed with 2% paraformaldehyde in phosphate buffered saline (PBS) for 15 min, then permeabilized with 0.25% Triton X-100. Next, cells were blocked with 5% goat serum, then incubated with 2 µg/mL of an affinity-purified rabbit polyclonal antibody specific for NIS (Abcam, Cambridge, MA) or rabbit anti-tuberin (C-20, Santa Cruz, CA) in PBS containing 1% nonfat dried milk, for 1 h at room temperature. Cells were then thoroughly washed with PBS and stained with 1 µg/mL of a Cy3-conjugated donkey anti-goat or anti-rabbit IgG (Jackson ImmunoResearch Laboratories Inc., West Grove, PA) for 1 h in the dark, washed with phosphate-buffered saline supplemented with 0.05% Tween-20 (PBS-T) and 4',6-diamidino-2-phenylindole (DAPI) for 5 min, and mounted with Fluoromount-G for observation under fluorescence microscopy. For immunohistochemistry, serial sections of tissues were fixed with 2% paraformaldehyde and blocked with 5% goat serum and permeabilized with 0.25% Triton X-100 as described above before being incubated with rabbit anti-GFP polyclonal antibody (1:200; Abcam), rabbit anti-NIS (1:50, Abcam), or rabbit anti-Ki-67 (1:50, Santa Cruz), and Alexa Fluor 680 (Invitrogen, Carlsbad, CA) goat anti-rabbit IgG (H+L) (1:200). TUNEL staining was performed using a TACS® 2 TdT-DAB *In Situ* Apoptosis Detection kit according to the manufacturer’s instructions (Trevigen, Inc., Gaithersburg, MD). Prior to TUNEL staining, tissue sections were rehydrated successively in 100%, 95%, and 70% ethanol, washed with PBS, and digested with proteinase K (1:100 dilution; Trevigen) for 1 hour at room temperature. Following quenching with 3% (vol/vol) hydrogen peroxide in methanol, the TUNEL reaction was performed for 1 h at 37°C and stopped with Stop Buffer (Trevigen) for 5 min. After 2 washes with deionized water, the reaction samples were incubated with 50 µl of Strep-HRP solution for 10 min at 37°C. After removing the solution and washing twice with PBS, the samples were then immersed in DAB solution (Trivigen) for 5 min. After several washes with deionized water, Methyl Green (Trivigen) counterstaining was performed according to the standard method.

**Delivery of TSC2-deficient cells to the lung airspaces via tracheal intubation:** Animal studies were performed in accordance with the approved institutional protocol #155-2008 by the Institutional Animal Care and Use Committee (IACUC) of Beth Israel Deaconess Medical Center. Female and male athymic NCr *nu/nu* mice were purchased from Taconic Farms (Hudson, NY). At the time of tumor cell inoculation, mice averaged 6 to 8 weeks of age and weighed 25 g ± 3 g. For tumor cell inoculation, anesthesia was induced using intraperitoneal (IP) injection of a mixture of 50-mg/kg ketamine hydrochloride (Ketaject; Phoenix Pharmaceutical Inc., St. Joseph, MO) and 5-mg/kg xylazine hydrochloride (Bayer Corp., Shawnee Mission, KS). Approximately 5 million 621-327 cells suspended in PBS to produce a total of 100 µL were delivered through the mouth, larynx, and vocal cords and into the trachea using a 20-gauge, 1.5-inch plastic cannula (B. Braun Melsungen AG, Germany) and a 1cc syringe.

Intratracheal instillation to the lung airspaces was performed as follows: (1) The animal was positioned against an angled restraining stand; (2) the mouth was opened to properly view the epiglottis (a speculum can be used to hold the mouth open, which facilitates the instillation procedure); (3) a cannula was inserted into the mouth and placed between the vocal cords and into the lumen of the trachea; (4) once the cannula had reached the right position, the cells (approximately 10 million in 100-µl PBS) were instilled immediately; (5) after waking from anesthesia and exhibiting normal behavior, the mice were placed back in the animal holding room.

**SPECT/CT imaging of LAM/TSC tumors *in vivo*:** LAM/TSC tumors were assessed on the days indicated using micro SPECT/CT. Two hours prior to imaging, animals were anesthetized with 2% isoflurane/balance O_2_ and 500 µCi of ^99m^TcO_4_^-^ in 50-μl saline, which was injected intravenously. For imaging, animals were anesthetized with 2% isoflurane/balance O_2_ and scanned on a NanoSPECT/CT (Bioscan Inc., Washington, DC) equipped with an 8W X-ray source running at 65 kV (123 mA), and a 48-µm-pitch CMOS-CCD X-ray detector. Continuous helical microCT scanning was employed with the following parameters: 1 s exposure, 240 angles, 1.3 magnification, 37 mm pitch (1 field of view), and a 512 x 256 pixel frame size (192 µm pixels). Images were reconstructed as 170 x 170 pixel transverse matrices with varying axial length and slice thickness of 0.4 mm (isotropic voxel size 0.4 mm) using filtered-back projection (SheppLogan filtering).

Helical micro SPECT was performed using a four-headed gamma camera outfitted with multi-pinhole collimators having 2.5-mm diameter pinholes (36 total). Images were acquired over 360˚ in 48 projections of 50 s each using a 256 x 256 frame size (1.0 mm pixels). The micro-SPECT images were reconstructed as 86 x 86 pixel transverse matrices with varying axial length and slice thickness of 0.8 mm (isotropic voxel size 0.8 mm). Quantitation of tumor volume, and ^99m^Tc update was performed using InVivoScope™ software (Bioscan Inc., Washington, DC).

**Tumor measurements:** The “hot spot” tumor diameter in the maximum intensity projection (MIP) SPECT images was measured and compared to the physical size of tumors measured after animal sacrifice using calipers (Supplementary Figure 1). As expected, acquisition and reconstruction settings on the SPECT instrument designed to maximize sensitivity lead to a consistent and linear overestimate of the actual tumor size.

**GFP fluorescence imaging *in vivo* and *ex vivo*:** Visible fluorescence data were collected using the Maestro™ (Cambridge Research & Instrumentation, Inc., Woburn, MA) *in vivo* imaging system as previously described [3]. For GFP fluorescence, animals were imaged for fluorescence emission from 500 to 720 nm using a blue excitation filter (445 to 490 nm) and a 515-nm long-pass secondary emission filter.

**Image-guided resection of tumors:** LAM/TSC tumors and control lymph nodes were resected under SPECT/CT guidance and analyzed *ex vivo* (Supplementary Figure 2).

**Identification of TSC2 genetic mutations in tumors and organs:** Tumors and organs were excised immediately after animal sacrifice, embedded in Tissue-Tek OCT, flash-frozen in LN_2_, and stored at -80°C until use. Of note, tumor cells were not micro-dissected away from normal tissue. For a positive control, genomic DNA was isolated from 621-327 cells using a QIAamp® DNA Mini Kit (Qiagen GmbH, Hilden, Germany). The manufacturer’s protocol for DNA purification from cultured cells was followed. Genomic DNA from tumor-bearing mice was used as a template to amplify exon 17 of the TSC2 gene (of note, the previous exon 16 is now termed “exon 17”). The polymerase chain reaction (PCR) mixture, in a total volume of 25 µL, contained 50-µM deoxynucleoside triphosphates, 1.0-U Taq DNA polymerase, 1.5-mM MgCL2, 1 x PCR buffer, and a primer pair at 1 µM. The primers used for amplification (product sizes are in parentheses) are as follows:

First primer set (366 bp):

First forward primer (genomic sequence 26088-26108):

5' GTGCTGTCTTAGGACTGCGTT 3'

First reverse primer (genomic sequence 26343-26363):

5' GTCTCTGCAGCTTCCAGGAAC 3'

Second primer set (252 bp):

Second forward primer (genomic sequence 26101-26121)

5’ ACTGCGTTTTCACCTCCTGCG 3’

Second reverse primer (genomic sequence 26329-26351):

5’ TCCAGGAACCACACCTGCCGAGA 3’

Sequencing primers:

Forward:

5' ACTGCGTTTTCACCTCCTGCG 3’ or

5' ACCAAGCTGTACACCCTG CCTG 3’

Reverse:

5' CTGGCTGGGCCCCCAGGAACTG 3'

For PCR, the reaction mixture was initially denatured at 95°C for 5 min and then subjected to 35 cycles of 94°C for 15 s, 58°C at 30 s, and 70°C for 60 s. After final extension at 70°C for 7 min, products were separated on a 2.5% agarose gel along with base-pair markers, visualized with ethidium bromide, and quantified for band intensity. If no single band was seen on the gel, then nested PCR was performed using the second set primers and running the same cycles as mentioned above. The PCR-amplified exon 17 products for 621-327 cells alone (Supplementary Figure 3) and mouse tissue and tumors (Supplementary Figure 4) by DNA sequencing using the sequencing primers. Of note, the wild-type mouse *TSC2* gene has a C in the location of the human mutation.

**Effect of rapamycin on NIS-mediated radiotracer uptake:** To determine whether continuous exposure to rapamycin affected ^99m^TcO_4_^-^ uptake by NIS, NIS/GFP expressing 621-327 cells (5 x 10^6^ cells) and control 621-101 cells (5 x 10^6^ cells) were in incubated with 10 nM rapamycin at 37ºC for 24 h. 250 µCi of ^99m^TcO_4_^-^ was then added and the cells incubated an additional 1 h at 37˚C prior to quantitation. To determine whether rapamycin exerted any immediate effects on NIS uptake, the experiment was repeated using a 1 h exposure to rapamycin. Control cells were exposed to vehicle only. Results are summarized in Supplementary Figure 5 and suggested that there was no measurable effect of rapamycin on NIS-mediated uptake of ^99m^TcO_4_^-^.

**Proliferation and apoptosis of LAM/TSC tumors 2 weeks after ending rapamycin treatment:** Immediately after SPECT/CT imaging at 2 weeks post-treatment, mice were sacrificed. Tumors and organs were excised and immediately embedded in Tissue-Tek O.C.T., flash-frozen in LN_2_, and stored at -80°C until use. The rates of cell apoptosis and proliferation in the lung tissue were assessed on frozen lung sections using a TACS® 2 TdT-DAB *In Situ* Apoptosis Detection kit and rabbit anti-Ki-67 antibody (Supplementary Figure 6).

**Statistical Analysis:** All experiments were repeated independently at least twice. A Student's *t* test was used to examine the differences between the experimental groups.

**SUPPLEMENTARY REFERENCES**

1. Luo J DZ, Luo X, Tang N, Song WX, Chen J, Sharff KA, Luu HH, Haydon RC, Kinzler KW, Vogelstein B, He TC. (2007) A protocol for rapid generation of recombinant adenoviruses using the AdEasy system. Nat Protoc 2: 1236-1247.

2. Yu J, Astrinidis A, Howard S, Henske EP (2004) Estradiol and tamoxifen stimulate LAM-associated angiomyolipoma cell growth and activate both genomic and nongenomic signaling pathways. Am J Physiol Lung Cell Mol Physiol 286: L694-700.

3. Liu F, Bloch N, Bhushan KR, De Grand AM, Tanaka E, et al. (2008) Humoral bone morphogenetic protein 2 is sufficient for inducing breast cancer microcalcification. Mol Imaging 7: 175-186.
